# Novel association of eosinophilic pustular folliculitis with systemic lupus erythematosus: A case report

**DOI:** 10.1016/j.jdcr.2024.08.012

**Published:** 2024-09-02

**Authors:** Tyler B. Cepica, Rohit Gupta, Kaveh Nezafati

**Affiliations:** Department of Dermatology, University of Texas Southwestern Medical Center, Dallas, Texas

**Keywords:** basophils, dermatosis, eosinophilic pustular folliculitis, eosinophils, EPF, SLE, systemic lupus erythematosus

## Introduction

Eosinophilic pustular folliculitis (EPF) is a rare inflammatory dermatosis characterized by chronic, recurrent, and pruritic papulopustular eruptions that, on histology, demonstrate folliculotropic infiltrates of eosinophils frequently accompanied by eosinophilic micro-abscess.^1^

EPF can be subdivided into 3 clinical variants: classic EPF, EPF of infancy, and immunosuppression-associated EPF (IS-EPF). Classic EPF is predominantly seen in middle-aged, East-Asian patients who are otherwise healthy.^2^ EPF of infancy occurs around 6 months of age with a predilection for males and scalp involvement.^3^ IS-EPF has been primarily described in patients with HIV or hematologic malignancies.^4^^,^^5^ Lesions of IS-EPF tend to lack an annular arrangement and predilection for the face.^2^

Systemic lupus erythematosus (SLE) is a systemic autoimmune disease with potential for widespread organ involvement.^6^ EPF has been reported in a variety of conditions, including asthma, pregnancy, and hepatitis C, but has not yet been reported in SLE.^2^ We report 2 cases of IS-EPF in patients with SLE and propose a mechanistic explanation for the 2 conditions coexisting.

## Case 1

A 43-year-old Puerto Rican male presented with a 2-year history of red bumps on his face, neck, trunk, and extremities. Lesions were intensely pruritic, remitted and recurred, and had no clear trigger. Fifteen years prior, the patient had been diagnosed with SLE. He was not receiving systemic therapy for his SLE at the time of lesion onset and, according to the Systemic Lupus Erythematosus Disease Activity Index 2000, had active systemic disease (inflammatory arthritis, inflammatory rash, decreased complement component 4 [10 mg/dL; reference range [r.r.], 15-57)], and positive double-stranded DNA antibodies [1:40]).^7^

Previously, for this eruption, the patient had received several oral and topical medications including topical triamcinolone, topical tacrolimus, oral dapsone, oral minocycline, oral doxycycline, and oral azathioprine, none of which had significant benefit. Notable past treatments with some therapeutic efficacy included prednisone, indomethacin, and mycophenolate mofetil.

**Fig 1:**
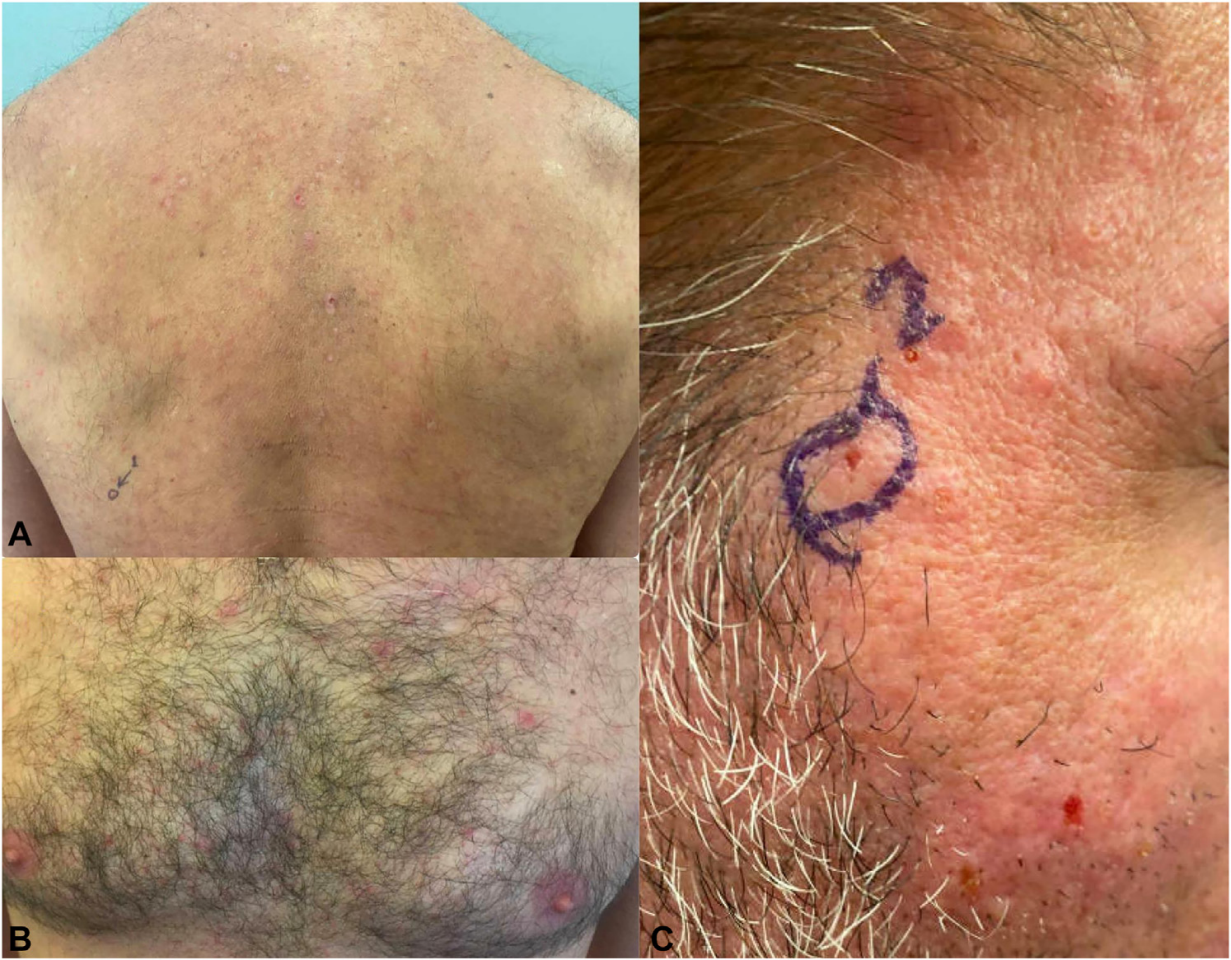
Initial presentation of eosinophilic pustular folliculitis in a 43-year-old Puerto Rican male showing several *pink* papules and pustules, some excoriated, along the patient’s (**A**) upper back (**B**) chest and (**C**) face, including the right temple and upper cheek.

**Fig 2:**
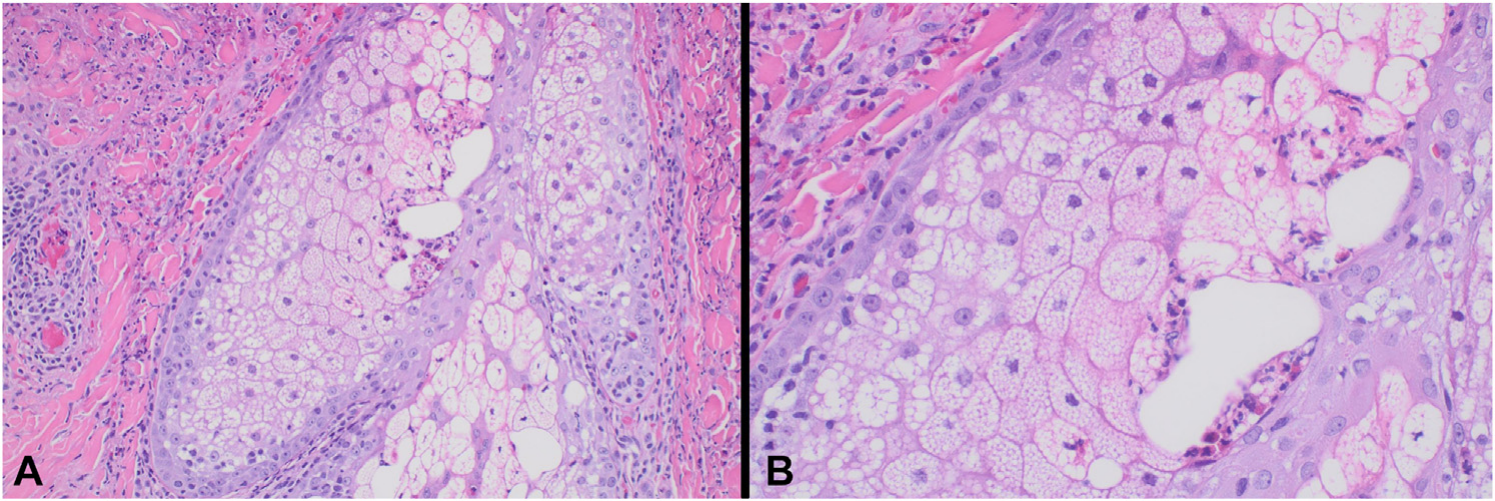
Histologic evaluation of a punch biopsy of the left upper back depicting eosinophils and neutrophils in and around the pilosebaceous unit, confirming the diagnosis of eosinophilic pustular folliculitis (hematoxylin and eosin; (**A**) 200×; (**B**) 400×). Immunohistochemical staining (not depicted) revealed CD3-positive perifollicular lymphocytes with no epitheliotropism, an increased CD4:CD8 ratio, and retention of CD7 positivity.

At the time of presentation, physical examination revealed several pink folliculocentric papules and pustules, some coalescing into plaques, scattered diffusely along the patient’s face, upper back, chest, arms, and upper thighs (Fig 1). Punch biopsies revealed moderately dense infiltrate consisting of lymphocytes, neutrophils, and eosinophils surrounding a dermal pilosebaceous unit (Fig 2). Periodic Acid-Schiff stain revealed no fungal elements.

Laboratory work-up revealed elevated serum immunoglobulin (Ig) E (12,050 kU/L; r.r. ≤214) and IgG (3760 mg/dL; r.r. 767-1590) with normal serum IgA (442 mg/dL; r.r. 63-484), IgM (71 mg/dL; r.r. 22-240), and absolute eosinophils (20 cells/uL; r.r. 0-700). HIV, hepatitis B and C, and work-up for hematologic and solid organ malignancies were negative. Other positive auto-immune antibodies included antinuclear antibody (1:1280) and anti-Ro (>8.0 U; r.r. <1.0). Fungal cultures and stool ova and parasite studies were negative.

Correlation of clinical presentation, laboratory data, and histopathology was consistent with a diagnosis of IS-EPF. The patient has been subsequently treated with oral isotretinoin, oral acitretin, and topical tacrolimus and triamcinolone. The greatest reduction in papulopustular burden and pruritus has been noted with oral isotretinoin.

## Case 2

**Fig 3:**
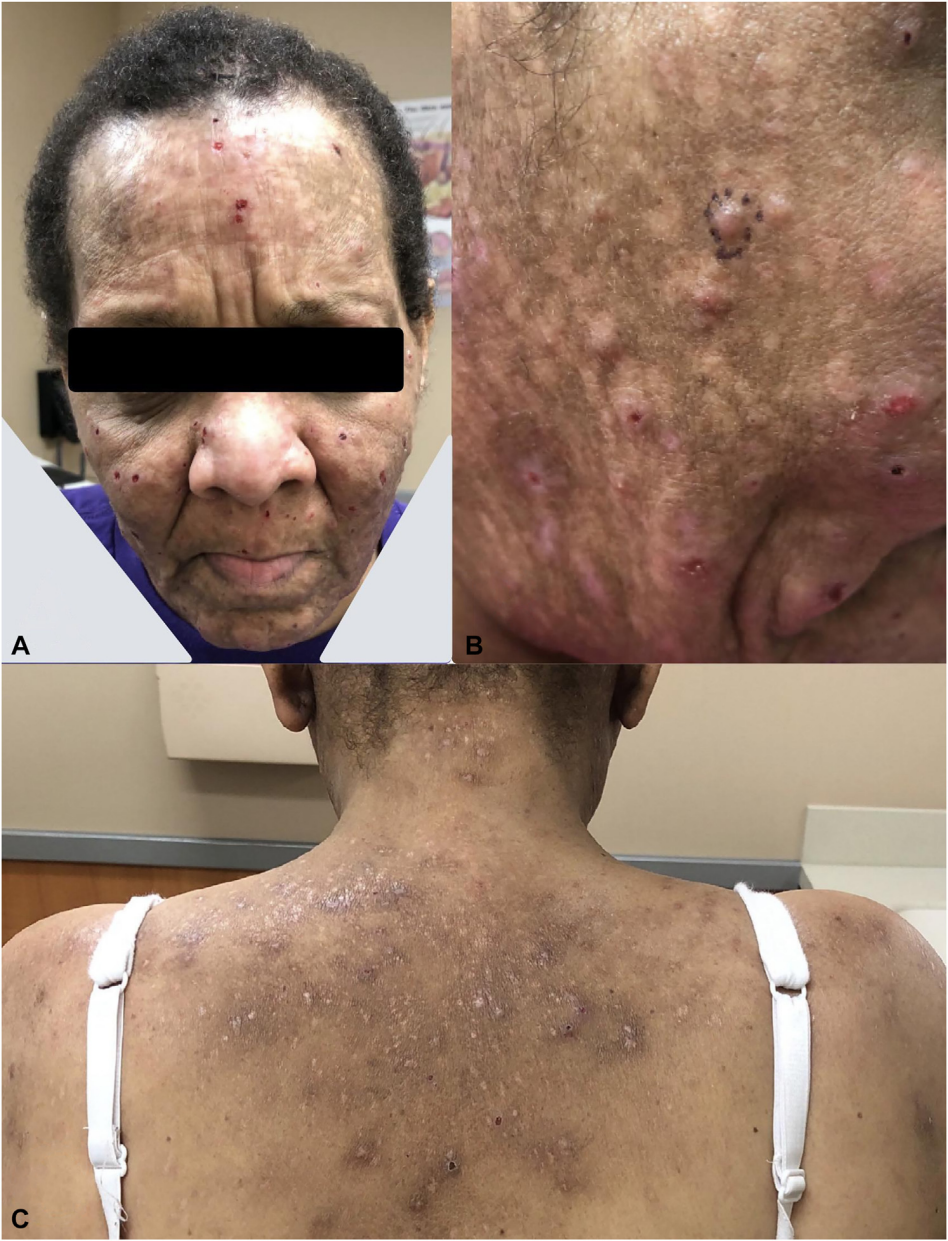
Initial presentation of eosinophilic pustular folliculitis in a 52-year-old Black female showing scattered erythematous papules and pustules, some excoriated, with background dyspigmentation and scarring on the (**A**) face, including the forehead, lateral nose, cheeks, and upper lip; (**B**) right lateral cheek and jaw; (**C**) posterior neck, upper back, and shoulders.

A 52-year-old black female with a history of SLE, asthma, and allergic rhinitis presented with a 3-year history of pruritic papules and pustules on her face, scalp, upper back, and chest (Fig 3). Past treatments for her eruption included doxycycline, ivermectin, permethrin, gentamicin, and mupirocin; none resulted in significant improvement. The patient was diagnosed with SLE 25 years prior, was not on any systemic medications for SLE, and, according to the Systemic Lupus Erythematosus Disease Activity Index 2000, had active systemic disease (inflammatory arthritis, inflammatory rash, alopecia, decreased complement component 3 [85 mg/dL; r.r. 90-180], and thrombocytopenia [93 × 10^9^/L; r.r. 160-383 × 10^9^]).

A punch biopsy of the cheek demonstrated nodular infiltrate of lymphocytes, histiocytes, and eosinophils centered around a follicle. Immunohistochemical staining revealed CD3-positive lymphocytes with few scattered CD20-positive cells. CD30-positivity fell short of that seen in true CD30-positive lesions.

Laboratory work-up revealed elevated serum IgE (40,425 kU/L; r.r. ≤114), IgG (2410 mg/dL; r.r. 600-1640), and absolute eosinophils (1161 cells/uL; r.r. 50-500) with normal levels of IgA (226 mg/dL; r.r. 47-310) and IgM (60 mg/dL; r.r. 50-300). HIV and hepatitis A, B, and C work-up was negative. Positive auto-immune antibodies included antinuclear antibody (1:2560), anti-ribonucleoprotein (5.0 AI; r.r. 0.0-0.9), and anti-Ro (2.2 AI: r.r. 0.0-0.9). Malignancy and infectious workup was negative. Correlation of clinical presentation, laboratory work-up, and histopathology was consistent with IS-EPF. The patient has since been prescribed tacrolimus, clobetasol, triamcinolone, prednisone, and belimumab, but has seen minimal improvement.

## Discussion

As a rare dermatosis, the pathogenesis of IS-EPF remains largely unknown. Though eosinophils play a critical role in the pathomechanism of EPF, basophils have also emerged as important factors in disease pathogenesis.^2^ Basophils express chemoattractant receptor-homologous molecule expressed on T-helper type 2 cells, a prostaglandin D2 (PGD2) receptor and an important contributor in eosinophil chemoattraction.^2^ Furthermore, IgE has been elevated in several cases of EPF and has been shown to bind to basophils and promote eosinophil tissue entry in IgE-dependent dermatitis.^2^^,^^8^

Recent studies have also revealed that IgE may play a critical role in the pathomechanism of SLE. In particular, autoreactive IgE functions to promote production of tumor necrosis factor-alpha via plasmacytoid dendritic cells and stimulating basophils, resulting in increased auto-antibody production.^9^ Moreover, in murine models, the bispecific PGD2 receptor 1 and 2 antagonist, AMG853, resulted in decreased biomarkers of lupus-like disease activity, implying that the PGD2-basophil interaction may be contributing to SLE disease.^10^

In both of the above patient cases, IgE was significantly elevated in the setting of patients presenting with EPF, comorbid SLE, and an otherwise negative work-up for other EPF-associated diseases. As such, elevations in IgE and a component of autoreactivity may be contributing to both the pathogenesis of SLE and EPF via the above-proposed mechanism. Furthermore, PGD2, though not measured directly in either patient, may play a synergistic role in both SLE and EPF disease.

In summary, we present 2 cases of IS-EPF and SLE, diseases in which IgE and PGD2 may both play unique roles. Further studies are needed to validate the proposed association between IS-EPF and SLE and potentially associations of IS-EPF with other conditions involving IgE and PGD2 in disease pathogenesis.

## Conflicts of interest

None disclosed.

## References

[1] Nomura T., Katoh M., Yamamoto Y., Miyachi Y., Kabashima K. (2016). Eosinophilic pustular folliculitis: a published work-based comprehensive analysis of therapeutic responsiveness. J Dermatol.

[2] Long H., Zhang G., Wang L., Lu Q. (2016). Eosinophilic skin diseases: a comprehensive review. Clin Rev Allergy Immunol.

[3] Fujiyama T., Tokura Y. (2013). Clinical and histopathological differential diagnosis of eosinophilic pustular folliculitis. J Dermatol.

[4] Afonso J.P., Tomimori J., Michalany N.S., Nonogaki S., Porro A.M. (2012). Pruritic papular eruption and eosinophilic folliculitis associated with human immunodeficiency virus (HIV) infection: a histopathological and immunohistochemical comparative study. J Am Acad Dermatol.

[5] Takamura S., Teraki Y. (2016). Eosinophilic pustular folliculitis associated with hematological disorders: a report of two cases and review of Japanese literature. J Dermatol.

[6] Kiriakidou M., Ching C.L. (2020). Systemic lupus erythematosus. Ann Intern Med.

[7] Gladman D.D., Ibañez D., Urowitz M.B. (2002). Systemic lupus erythematosus disease activity index 2000. J Rheumatol.

[8] Cheng L.E., Sullivan B.M., Retana L.E., Allen C.D., Liang H.E., Locksley R.M. (2015). IgE-activated basophils regulate eosinophil tissue entry by modulating endothelial function. J Exp Med.

[9] García-Carrasco M., Macias-Díaz S., Mendoza-Pinto C. (2021). The role of IgE in systemic lupus erythematosus. Autoimmun Rev.

[10] Pellefigues C., Tchen J., Saji C., Lamri Y., Charles N. (2022). AMG853, A bispecific prostaglandin D(2) receptor 1 and 2 antagonist, dampens basophil activation and related lupus-like nephritis activity in lyn-deficient mice. Front Immunol.

